# One Extinct Turtle Species Less: *Pelusios seychellensis* Is Not Extinct, It Never Existed

**DOI:** 10.1371/journal.pone.0057116

**Published:** 2013-04-03

**Authors:** Heiko Stuckas, Richard Gemel, Uwe Fritz

**Affiliations:** 1 Museum of Zoology, Senckenberg Dresden, Dresden, Germany; 2 Natural History Museum Vienna, Vienna, Austria; Tuscia University, Italy

## Abstract

*Pelusios seychellensis* is thought to be a freshwater turtle species endemic to the island of Mahé, Seychelles. There are only three museum specimens from the late 19^th^ century known. The species has been never found again, despite intensive searches on Mahé. Therefore, *P. seychellensis* has been declared as “Extinct” by the IUCN and is the sole putatively extinct freshwater turtle species. Using DNA sequences of three mitochondrial genes of the historical type specimen and phylogenetic analyses including all other species of the genus, we provide evidence that the description of *P. seychellensis* was erroneously based on a widely distributed West African species, *P. castaneus*. Consequently, we synonymize the two species and delete *P. seychellensis* from the list of extinct chelonian species and from the faunal list of the Seychelles.

## Introduction

Among the approximately 320 recognized species of turtles and tortoises [1]–[4], some are thought to be extirpated by direct or indirect impact of humans, and all confirmed cases refer to island taxa [2], [5]. The most spectacular case is *Meiolania damelipi*, the last representative of the famous giant ‘horned turtles’ (Meiolaniidae) that disappeared from Efate island (Vanuatu, Southwest Pacific) approximately 3000 years before present, within 300 years after the arrival of humans [6]. With this species, a major evolutionary lineage of turtles [7] vanished for ever from earth. Other well-known examples include different species of giant land tortoises (Testudinidae). The Malagasy tortoise species *Aldabrachelys abrupta* and *A. grandidieri* became extinct 750±370 and 1250±50 years before present, respectively, most likely as a consequence of the human colonisation of Madagascar [8]. Besides a few extinct or perhaps extinct taxa of giant Galápagos tortoises (*Chelonoidis nigra* complex [9]–[13]), also all five species of the Mascarene tortoise genus *Cylindraspis* [14] were evidently eradicated by sailors, settlers and introduced animals, like feral pigs and rats, between 1735 and 1840 [15]–[17]. In addition to these terrestrial species, there were only two freshwater turtle species thought to be extinct [18], [19]. However, some years ago live individuals of *Cuora yunnanensis* (Geoemydidae), the only putatively extinct non-island species, were discovered [20], so that there is currently only one freshwater turtle species considered extinct [2], [5], [21]: *Pelusios seychellensis* (Pelomedusidae), a species known from only three specimens collected on the island of Mahé, Seychelles, in the late 19^th^ century [22]–[25].

Currently, the genus *Pelusios* comprises 17 to 18 recognized species [2], [5], [26]. Yet, some recently identified, clearly divergent genetic lineages seem to represent further distinct species [26], [27]. *Pelusios* species are small- to medium-sized freshwater turtles that occur in sub-Saharan Africa, Madagascar and on the Seychelles [2], [5], [23], [28]. Their most conspicuous morphological character is the movable plastral forelobe, allowing the closure of the anterior shell opening [28]–[30]. Their common name, hinged terrapins, refers to this peculiarity.

**Figure 1:**
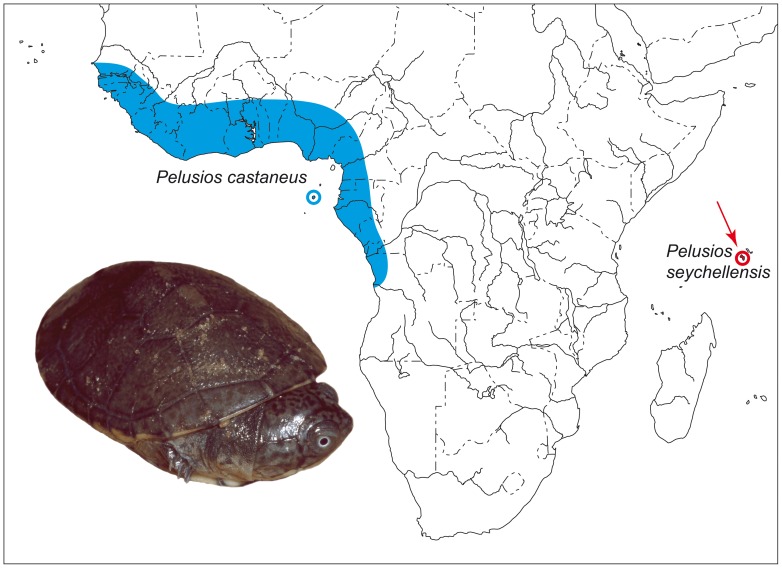
Distribution of *Pelusios seychellensis* (arrow) and *P. castaneus*. Introduced population of *P. castaneus* on Guadeloupe (Lesser Antilles) not shown. Inset: *P. castaneus* (hatchling, Comoé National Park, Ivory Coast).

The Seychelles are a mid-oceanic tropical archipelago of 115 islands, about halfway between Madagascar and India (Fig. 1). Some of these islands, the granitic Seychelles, are remnants of the supercontinent Gondwana and were ‘lost in the sea’ during the north-eastward rafting of India after its detachment from Africa, approximately 63.4 million years ago [31], [32]. Mahé, from where the putatively extinct *P. seychellensis* was described, is the main island of the granitic Seychelles. As a legacy of the breakup of Gondwana, the fauna of the Seychelles comprises many paleo-endemic species. There is also a number of younger endemics that arrived later by transoceanic dispersal; all endemic species are genetically clearly distinct [32]–[36]. Considering their small surface and the paucity of suitable freshwater habitats (less than 11 ha [37]), the Seychelles harbour an extraordinarily rich fauna of freshwater turtles. There are three *Pelusios* species known from the Seychelles islands, *P. castanoides*, *P. subniger* and the extinct *P. seychellensis*. While *P. castanoides* and *P. subniger* occur on several islands, *P. seychellensis* was recorded only from Mahé [23], [25], [38], [39]. It has been speculated *P. seychellensis* is an ancient component of the Seychellois fauna and that *P. castanoides* and *P. subniger*, representing younger colonizers of the Seychelles, outcompeted *P. seychellensis* with increasing human pressure [25].

In 1983, Bour [23] resurrected *P. seychellensis* (Siebenrock, 1906) as a species endemic to Mahé (Fig. 1). In addition, Bour [23] described the Seychellois populations of *P. castanoides* and *P. subniger* as the new subspecies *P. c. intergularis* and *P. s. parietalis*, endemic to the Seychelles. However, a recent molecular genetic investigation provided firm evidence that at least *P. subniger* was introduced to the Seychelles, and this possibility could also not be ruled out for *P. castanoides* [27], fuelling the hypothesis of niche competition between an endemic species and naturalized freshwater turtles.

Starting with its original description by Siebenrock in 1906 [22], several authors having studied *P. seychellensis* were explicitly puzzled by its morphological similarity to a West African *Pelusios* species, *P. castaneus* [22], [23], [25], [40], [41]. However, related to the complicated taxonomic and nomenclatural history of the genus *Pelusios*, other authors [42], [43] confused *P. seychellensis* with another superficially similar species occurring on the Seychelles (*P. castanoides*). As a consequence, *P. seychellensis* was soon synonymized with one or the other species [40], [43]–[45] and not recognized as distinct until Bour resurrected it in 1983 in his revision of Seychellois hinged terrapins [23]. Yet, in the face of the morphological similarity of *P. seychellensis* and *P. castaneus*, the possibility that *P. seychellensis* was founded on a mislabelled museum specimen of *P. castaneus* requires examination. There is quite a number of vertebrate species that were erroneously described using museum specimens of well-known species bearing incorrect locality data (e.g., [5], [46]–[52]), suggesting that this could be also the case in *P. seychellensis*.

Therefore, here we scrutinize the validity of *P. seychellensis* using historical DNA sequences of three mitochondrial genes (12S rRNA, cyt *b*, ND4), generated from its name-bearing lectotype. For doing so, we merge these data with previously published sequences of all other *Pelusios* species [26], [27], [53] and analyse this 2054-bp-long data set using Bayesian and Maximum Likelihood methods. Owing to the long independent history of the Seychelles, *P. seychellensis* should be clearly distinct from the 16 or 17 currently recognized other species of the genus.

## Materials and Methods

### Chosen markers and primer design

Using mitochondrial DNA sequences, all currently known species and genetic lineages of *Pelusios* are phylogenetically clearly distinct and unambiguously identifiable. Previous studies [26], [27], [53] relied on partial sequences of the small subunit of ribosomal RNA (12S), the cytochrome *b* gene (cyt *b*), and another mitochondrial DNA fragment, corresponding to the second half of the NADH dehydrogenase subunit 4 gene (ND4) plus adjacent DNA coding for tRNAs. Therefore, we aimed at reconstructing homologous sequence data for the name-bearing lectotype of *P. seychellensis*. Considering the morphological similarity of *P. seychellensis* and *P. castaneus*, DNA sequences of the latter species served as template for primer design. As *P. castanoides*, one of the two extant *Pelusios* species occurring on the Seychelles islands, is also superficially similar in morphology, primers were designed having an optimal fit to DNA sequences of both species using previously published data sets [26], [27], [53]. The resulting amplicons were of approximately 150–200 bp length.

While the 12S primers successfully amplified two sequences of 178 bp and 164 bp (overlap 36 bp), our initial efforts failed for many DNA fragments of similar length for the two other genes. Therefore, new primer pairs were designed that yielded shorter non-overlapping DNA fragments, again with sequences of *P. castaneus* and *P. castanoides* as templates. Then, the obtained DNA sequences were used to design specific primer pairs to fill the gaps with overlapping sequences. Obtained DNA fragments were of 28–130 bp (cyt *b*) and 62–176 bp length (ND4 + tRNAs) after primer sequences were trimmed and overlapped by 14–66 bp (cyt *b*) and 15–72 bp (ND4 + tRNAs).

### The lectotype, DNA extraction, PCR and sequencing

**Figure 2:**
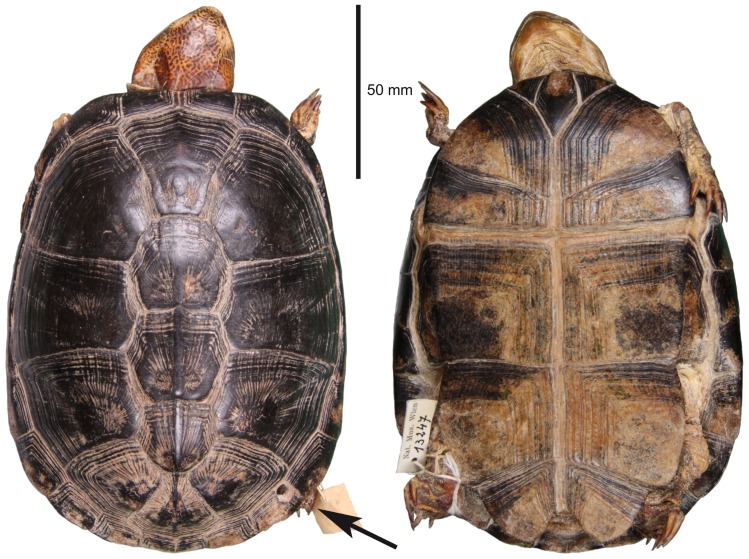
Dorsal (left) and ventral aspect (right) of the lectotype of *Pelusios seychellensis*. Female, Natural History Museum Vienna, Austria (NHMW 13247). The arrow indicates the hole drilled in the shell margin (see text).

The lectotype of *Pelusios seychellensis* is a dry specimen of 126 mm straight-line carapacial length (Fig. 2). This young female is preserved in the herpetological collection of the Natural History Museum Vienna, Austria, and registered under catalogue number NHMW 13247. It was obtained between 1901 and 1906 from the Zoological Museum Hamburg and is thought to be collected on the island of Mahé, Seychelles, by August Brauer in 1895 or 1896 (see Discussion). For obtaining DNA, dried muscle tissue was removed from the thigh region close to the femoral bone by carefully opening the skin using sterile equipment.

DNA was extracted in a clean room using a HERAsafe KSP 9 safety cabinet (Thermo Fisher Scientific; Waltham, MA, USA) and the sbeadex forensic kit (LGC Genomics; Berlin, Germany) according to the standard protocol recommended by the supplier. This clean room is physically isolated from all other DNA processing facilities, and no *Pelusios* samples have been studied there before (fresh *Pelusios* samples for previous studies [26], [27], [53] were processed in the main laboratory, which is located in another building). Also the PCR setup for the lectotype was performed in a laminar flow cabinet of the clean room, using a final volume of 25 µl containing 1 unit *Taq* polymerase (Bioron; Ludwigshafen, Germany) with the buffer recommended by the supplier, a final concentration of 4 mM MgCl_2_ (Bioron), 0.2 mM of each dNTP (Fermentas; St. Leon-Rot, Germany), 0.4 µM of the respective primer (Table S1), and 0.4 µg of Bovine Serum Albumin (Fermentas). Workstations and clean room were irradiated with UV light at least 6 h before and after every working step.

Thermocycling was carried out in the main laboratory, and a positive control (containing DNA of a fresh *P. castaneus* sample, extracted with standard methods in the normal DNA processing facility) and a negative control (all reagents except the DNA template) were always processed downstream along with the type samples. The following cycling conditions were used for amplification of all PCR products: 40 cycles with denaturation at 95°C for 45 s, but for 5 min in the first cycle; annealing for 45 s at the primer-specific temperature (Table S1); and extension at 72°C for 1 min, but for 10 min in the final cycle. PCR products were purified using the ExoSAP-IT enzymatic cleanup (USB Europe GmbH; Staufen, Germany; 1∶20 dilution, modified protocol: 30 min at 37°C, 15 min at 80°C) and sequenced on an ABI 3130xl Genetic Analyser (Applied Biosystems; Foster City, CA, USA) using the same primers (Table S1) and the BigDye Terminator v3.1 Cycle Sequencing Kit (Applied Biosystems). However, the cyt *b* fragment obtained from the primer combination cytB_for12 and cytB_rev12 could be not sequenced completely using these primers, so that an internal primer pair was designed to bridge the missing sites (cytB_for12seq: CTC CGA AAT TTA CAC GCT A, cytB_rev12seq: ATG ACA CCC GTG TAT CAA).

### Alignment and phylogenetic analyses

The obtained type sequences were aligned in BIOEDIT 7.0.5.2 [54]. Uncorrected pairwise distances (pairwise deletion) for contig sequences of the type, the positive control and some other sequences were calculated with MEGA 5.05 [55]. Contigs for the 12S, cyt *b* and ND4 sequences of the type were merged with the previously published data sets for *Pelusios* [26], [27], [53]. Like in Fritz et al. [26], the nine deeply divergent lineages of *Pelomedusa* [56] and *Podocnemis expansa* were included as outgroups, resulting in an alignment of 2054 bp. The *Pelomedusa* lineages together constitute the sister group of *Pelusios* [26], and *Podocnemis expansa* represents among extant turtles the successive sister taxon, the Podocnemididae [57]. For calculations, identical sequences were removed from the alignment. The partitioning scheme (Table S2) was the same as in Fritz et al. [26].

Phylogenetic relationships were inferred using MRBAYES 3.2.1 [58], with the best evolutionary model for each partition established in JMODELTEST 0.1.1 [59] using the Bayesian Information Criterion (Table S2). Evolutionary models were defined by giving the number of substitution types (nst) and the substitution rates for each partition, but without setting priors for alpha-parameter, points of invariance and rate matrix, allowing these parameters to be estimated by MRBAYES. Phylogenetic analyses were performed using two parallel runs (each with four chains) and the heating parameter λ set to 0.1. Both chains ran for 10 million generations with every 500^th^ generation sampled and reached a split frequency below 0.05. The calculation parameters were analysed using a burn-in of 2.5 million generations to assure that both chains converged. Subsequently, only the plateau of the most likely trees was sampled using the same burn-in and used for generating a 50% majority rule consensus tree. The posterior probability of any individual clade in this consensus tree corresponds to the percentage of all trees containing that clade, and is a measure of clade frequency and credibility.

In addition to Bayesian analyses, Maximum Likelihood (ML) analyses were conducted with RAxML 7.2.6 [60] and the implemented evolutionary model GTR+G. Five independent ML searches were performed using different starting conditions and the fast bootstrap algorithm to explore the robustness of the phylogenetic trees by comparing the best trees. Subsequently, 1000 non-parametric thorough bootstrap replicates were calculated and the values plotted against the best tree.

### Publication and nomenclatural act

This published work has been registered in ZooBank, the online registration system for the ICZN. The ZooBank LSID (Life Science Identifier) can be resolved and the associated information viewed through any standard web browser by appending the LSID to the prefix “http://zoobank.org/”. The LSID for this publication is: urn:lsid:zoobank.org:pub:0A43BDC6-2690-4288-AAFB-A9052B89FA36. The electronic edition of this work was published in a journal with an ISSN, and has been archived and is available from the following digital repositories: PubMed (http://www.pubmedcentral.nih.gov/) and LOCKSS (http://www.lockss.org/lockss/).

## Results

The short DNA sequences of the lectotype of *Pelusios seychellensis* were of good quality and easily readable. The resulting contig sequences (accession numbers HF559227-HF559229) were 306 bp (12S), 695 bp (cyt *b*) and 762 bp (ND4 + tRNAs) long. The negative controls never yielded DNA sequences, while the contig sequences obtained from the positive controls agreed with previously published GenBank sequences of the same sample (accession numbers FR716849, FR716906-08, FR716959). These sequences were weakly, but consistently different from the type sequences. Pairwise differences were distributed over all three contigs, providing evidence that we amplified and sequenced authentic DNA of the lectotype and not contaminant DNA. Pairwise differences between the type and the positive control were in the 12S fragment 1.0%, in the cyt *b* fragment 2.2% and in the fragment comprising ND4 + tRNAs 1.8%.

**Figure 3:**
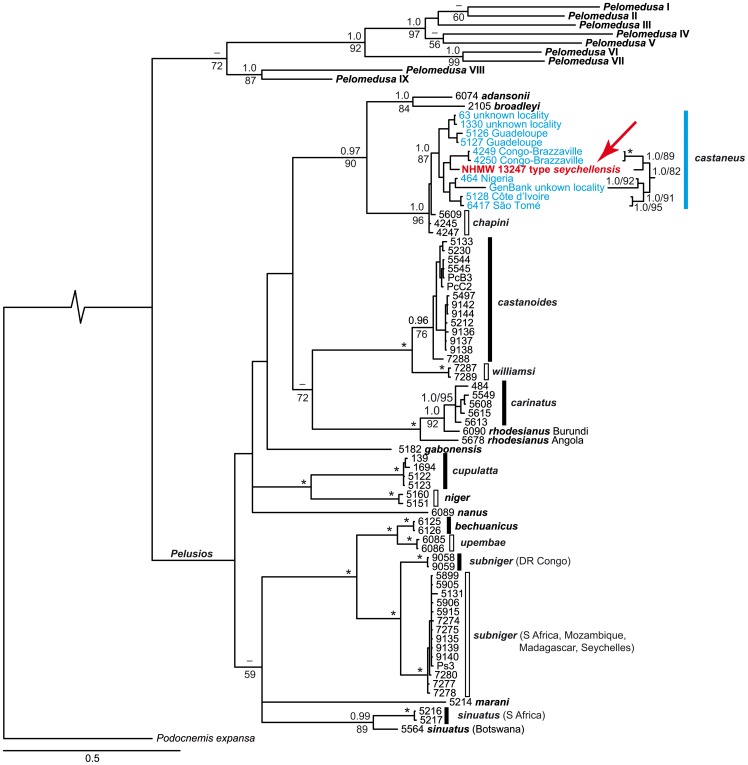
Phylogeny of *Pelusios* species, inferred from Bayesian analyses. Sample codes refer to the data sets of Fritz et al. [26], [27], [53], see there for further explanation. Numbers above branches are posterior probabilities greater than 0.95; numbers below branches are Maximum Likelihood bootstrap values greater than 50 (not shown for some terminal clades with short branches). Asterisks indicate maximum support under both methods. Root shortened by 80%. Note the placement of *P. seychellensis* among *P. castaneus* (arrow).

When the concatenated contigs of the lectotype were merged with the previously published data sets for *Pelusios* and *Pelomedusa* and used for phylogenetic inference, the tree topologies corresponded to those presented in Fritz et al. [26], [53]. All currently recognized *Pelusios* species, except *P. chapini* and *P. castaneus*, were clearly distinct, as were the previously identified lineages of *P. rhodesianus* (Burundi vs. Angola [26]), *P. subniger* (Democratic Republic of the Congo vs. remaining range [27]) and *P. sinuatus* (South Africa vs. Botswana [26]). The type specimen of *P. seychellensis* was embedded among West African *P. castaneus* and clustered, with high support, in this well-supported clade with two *P. castaneus* from Congo-Brazzaville (Fig. 3). The concatenated sequences of the latter two turtles differed from the lectotype on average by 1.2% pairwise divergence.

## Discussion

In the phylogenetic trees (Fig. 3), the DNA sequences of the name-bearing type specimen of *Pelusios seychellensis* are nested with high support among sequences of *P. castaneus*. This suggests that the two species are synonymous and that the description of *P. seychellensis* was erroneously founded on specimens of *P. castaneus*, a species which is morphologically so similar that even the describer of *P. seychellensis* questioned their distinctiveness. In the original description of the Seychellois taxon, Siebenrock [22] pointed out that “when the geographical separation between the two were not so colossal, there were the temptation to consider the two forms identical” (literally translated from the German), underlining that the distinction of the two species was mainly based on the geographical gap separating them (Fig. 1).

*Pelusios castaneus* is a West African species, distributed from Guinea and Senegal to the Central African Republic and northwestern Angola. It also occurs on the São Tomé islands and was introduced to Guadeloupe, Lesser Antilles [23], [28], [61]. Putative old records of the species on the islet of St. Maria near Praia (Cabo Verde) were never confirmed [62], [63], but continue to be repeated in recent literature [2]. The range of *P. castaneus* is far away from the Seychelles, being separated from those islands by the African landmass (Fig. 1). Therefore, it seems very unlikely that *P. castaneus* reached the Seychelles by natural means. This would necessitate either crossing the African continent and subsequent transoceanic dispersal to the Seychelles or transoceanic dispersal over more than 10,000 km, from the Atlantic to the Indian Ocean, circumventing en route the Cape of Good Hope and Madagascar. Consequently, there remain two alternative possibilities: Either the lectotype was mislabelled or it was transported by humans from West Africa to the Seychelles. Owing to the considerable distance between West Africa and the Seychelles, the latter option seems less likely.

The name-bearing lectotype of *P. seychellensis* and two further specimens of *P. seychellensis* are thought to be collected on Mahé, Seychelles, by August Brauer (3 April 1863 – 10 September 1917), a German zoologist who stayed from May 1895 to January 1896 on the island of Mahé [22], [23], [25], [64]. Brauer was stationed at Mamelles Plantation, near Port Victoria (Mahé), and undertook from there excursions into the mountains of Mahé and to other islands. After his return to Germany, Brauer joined the German Deep Sea Expedition (1898–1899) led by Carl Chun and went afterwards to Marburg, where he was appointed as a university professor in 1900. Six years later, Brauer became director of the Zoological Museum Berlin [64]. According to Bour & Gerlach [25] and surviving original labels, the specimens of *P. seychellensis* were acquired by the Zoological Museum Hamburg in 1901 from Brauer, that is, approximately five years after Brauer returned from the Seychelles, and two years after he returned from the German Deep Sea Expedition. It is possible that during this time specimens were confused in Brauer's private collection or that the turtles were mislabelled in the Hamburg museum. This suspicion is supported by the holes drilled in the marginal scutes of the lectotype (Fig. 2) and of at least one of the two other specimens of *P. seychellensis* (see the figure in Bour & Gerlach [25]). Such holes are used until today by marketers for tying captive turtles together until they are sold for food. This suggests that the lectotype and the other specimens of *P. seychellensis* were bought somewhere and that Brauer did not collect the turtles in the field.

The Viennese herpetologist Friedrich Siebenrock (20 January 1853–28 January 1925), then a leading authority in chelonian taxonomy [65], [66], studied the three putative Seychellois specimens in Hamburg and described in 1906, based on two specimens, the new taxon *Sternothaerus nigricans seychellensis* [22], which was synonymized by Siebenrock himself in 1916 [40]. About 70 years later, Bour [23] resurrected the taxon as a full species (*P. seychellensis*). Siebenrock obtained one specimen for the Natural History Museum in Vienna, which was later designated as the name-bearing lectotype [67]. The two other specimens, still in the Hamburg collection, are morphologically very similar to the lectotype, so that no doubt exists that they represent the same taxon [22], [23], [25]. Trials to generate DNA sequences from these specimens in another laboratory were unsuccessful (J. Hallermann, pers. comm.).

Following Bour's [23] recognition of *P. seychellensis* as a valid species in 1983, intensive searches on Mahé failed to locate any live specimens, leading to the assumption that the species is extinct [21], [25]. However, our genetic data provide evidence that this failure has another reason. The firm placement of the sequences of the lectotype of *P. seychellensis* among *P. castaneus* shows that *P. seychellensis* does not represent a distinct species. Rather, its description was founded on misidentified museum specimens of another species, *P. castaneus*, which is widely distributed in West Africa and which does not occur on Mahé, Seychelles (Fig. 1). Consequently, we place *P. seychellensis* into the synonymy of *P. castaneus* and delete *P. seychellensis* from the list of extinct chelonian species and from the faunal list of the Seychelles.

The case of *P. seychellensis* parallels two other turtle species that were described using museum specimens with incorrect locality data. Ogilby [68] described in 1905 *Devisia mythodes* as a genus and species new to science, based on a type specimen allegedly collected in the Fly River of New Guinea. Later, it turned out that this specimen is a North American snapping turtle (*Chelydra serpentina*), for which the locality data must have been confounded [46]. The case of *Testudo hypselonota*, a tortoise species described in 1941 from southern Vietnam [69], is slightly different in that the type specimen was collected in Vietnam indeed, but turned later out as an escaped pet tortoise from Madagascar (*Astrochelys radiata*) [47], [48].

With *P. seychellensis*, the Seychelles lose the second freshwater turtle species thought to be endemic there. Fritz et al. [27] have shown that the Seychellois populations of *P. subniger* do not represent a genetically distinct subspecies, as thought before [23], [39]. Rather, owing to completely lacking mitochondrial DNA differentiation, these turtles must have been introduced by humans from southeastern Africa. Consequently, there remains only one freshwater turtle species, *P. castanoides*, that could be native to the Seychelles islands [23], [27], [38]. However, also for *P. castanoides* doubts remain whether the weak mitochondrial DNA differentiation of the Seychellois populations reflects endemicity. Due to incomplete sampling from the species' range in continental Africa, it cannot be excluded that identical haplotypes occur somewhere else [27]. Even if *P. castanoides* should be native to the Seychelles, it is clear that there are distinctly less endemic freshwater turtle species on the archipelago than thought before [23], [25], [38], [39], and this insight should be implemented in future conservation and management strategies for the endangered native fauna of the Seychelles.

## Supporting Information

Table S1 **Primers used for PCR and sequencing of mtDNA fragments of the 12S, cyt** ***b*** **and ND4 genes (the latter including adjacent DNA coding for tRNAs).** First generation primers targeting conserved mtDNA regions of *Pelusios castaneus* and *P. castanoides* are indicated with asterisks; second generation primers to bridge non-overlapping DNA fragments, without asterisks.
(DOC)

Table S2 **Partitioning scheme and models selected by the Bayesian Information Criterion in JMODELTEST 0.1.1** [**59**] **.**
(DOC)
