# Supplementary material for: One Extinct Turtle Species Less: Pelusios seychellensis Is Not Extinct, It Never Existed
Source: PLoS One. 2013 Apr 3;8(4):e57116. doi: 10.1371/journal.pone.0057116 (PMC3616038; doi:10.1371/journal.pone.0057116)
Supplement: Table S1 — Primers used for PCR and sequencing of mtDNA fragments of the 12S, cyt b and ND4 genes (the latter including adjacent DNA coding for tRNAs). First generation primers targeting conserved mtDNA regions of Pelusios castaneus and P. castanoides are indicated with asterisks; second generation primers to bridge non-overlapping DNA fragments, without asterisks. (DOC) [file pone.0057116.s001.doc]

**Table S1.** Primers used for PCR and sequencing of mtDNA fragments of the 12S, cyt *b* and ND4 genes (the latter including adjacent DNA coding for tRNAs). First generation primers targeting conserved mtDNA regions of *Pelusios castaneus* and *P. castanoides* are indicated with asterisks; second generation primers to bridge non-overlapping DNA fragments, without asterisks.

| **Fragment** | **Primer** | **Primer sequence (5’–3’)** | **Annealing temperature [°C]** | **Length of obtained DNA fragment [bp]** |
| --- | --- | --- | --- | --- |
| 12S fragment 1 | 12S_for1* | AAT ATC CGC CAG AGA ACT A | 56 | 178 |
|  | 12S_rev1* | CCT GGA CCT GAC TTW CTA |  |  |
| 12S fragment 2 | 12S_for2* | TCT CCA GCT TAC CTT RT | 50 | 164 |
|  | 12S_rev2* | GCT TAA TYT AGG CTC TCT |  |  |
| cyt *b* fragment 1 | cytB_for1* | TCA CTA TCG CCT TCT CAT CAG | 56 | 96 |
|  | cytB_rev6* | GTA GTA GAT ACC TCG TCC AA |  |  |
| cyt *b* fragment 2 | cytB_for12 | CTC GAG ACG TCC AAT ACG | 56 | 126 |
|  | cytB_rev12 | TAT TGT TAR GAG YAG GAG GA |  |  |
| cyt *b* fragment 3 | cytB_for17 | GAC GAG GTA TCT ACT ACG | 56 | 95 |
|  | cytB_rev17 | GCA CCT CAA AAG GAT ATT TG |  |  |
| cyt *b* fragment 4 | cytB_for13* | ACA GCC TTT ATA GGR TAC | 60 | 48 |
|  | cytB_rev13* | CCY CCT CAG ATT CAT TGG |  |  |
| cyt *b* fragment 5 | cytB_for18 | TTA CCC TGA GGC CAA ATA TC | 56 | 130 |
|  | cytB_rev18 | ATG ATA AAA GGG GTC AGG AA |  |  |
| cyt *b* fragment 6 | cytB_for14 | AAC GCA ACC CTA ACY CGC | 60 | 106 |
|  | cytB_rev14 | GGT GAA ATG GGA TTT TGT CGC A |  |  |
| cyt *b* fragment 7 | cytB_for8* | AAT TCA CCT CTT ATT CCT YCA | 56 | 67 |
|  | cytB_rev8* | AGR AGG TCT TTG TAT GAG AA |  |  |

Table S1 continued

| **Fragment** | **Primer** | **Primer sequence (5’–3’)** | **Annealing temperature [°C]** | **Length of obtained DNA fragment [bp]** |
| --- | --- | --- | --- | --- |
| cyt *b* fragment 8 | cytB_for15 | AAC AGG CTC TAA TAA CCC | 56 | 88 |
|  | cytB_rev15 | GGT TGG GAT AGT TAG TCC |  |  |
| cyt *b* fragment 9 | cytB_for9* | AAC TCC GAC AAA ATC CCA TT | 56 | 90 |
|  | cytB_rev9* | TGA AGT TTT CAG GGT CTG TT |  |  |
| cyt *b* fragment 10 | cytB_for20 | CAT ACA AAG ACC TTC TAG G | 56 | 127 |
|  | cytB_rev20 | ATT GCG TAA GCA AAT AGG AA |  |  |
| cyt *b* fragment 11 | cytB_for16 | AAC CCT CCT CCT CCC YAA | 54 | 69 |
|  | cytB_rev16 | TAA CAC TCC TCC CAG TTT GTT |  |  |
| cyt *b* fragment 12 | cytB_for21 | CCC TCC ACA TAT CAA ACC | 56 | 82 |
|  | cytB_rev21 | GTA TGA GAA AAA GGA TAG TTA C |  |  |
| cyt *b* fragment 13 | cytB_for10* | GCC AAC CCC YTA ATC ACC | 60 | 36 |
|  | cytB_rev10* | GTT ACY GAG GCT AAG AGR G |  |  |
| cyt *b* fragment 14 | cytB_for11* | CGC TCC ATC CCA AAC AAA CT | 56 | 28 |
|  | cytB_rev5* | TTT GGG TTA GGG GTC GGA |  |  |
| ND4 fragment 1 | ND4_for6* | ATC CGA ATA ACC TTA ATM CTT A | 56 | 74 |
|  | ND4_rev6* | GTC TGT TTG TCG TAG GCA |  |  |
| ND4 fragment 2 | ND4_for16 | CCC TTC ATA GCC CTC TCT TT | 56 | 98 |
|  | ND4_rev16 | TGT TTG TAG GAG TGC TGC AG |  |  |
| ND4 fragment 3 | ND4_forN1 | TGY YTR CGH CAA ACA GAC | 51 | 151 |
|  | ND4_revN1 | RTG GGT YCG YTC RTA GTT TG |  |  |
| ND4 fragment 4 | ND4_forP2 | CAR CAC TCC TAC AAA CAC C | 56 | 73 |
|  | ND4_rev7 | GGG TYC GTT CRT AGT TTG TAT T |  |  |

Table S1 continued

| **Fragment** | **Primer** | **Primer sequence (5’–3’)** | **Annealing temperature [°C]** | **Length of obtained DNA fragment [bp]** |
| --- | --- | --- | --- | --- |
| ND4 fragment 5 | ND4_for2* | CAR CAC TCC TAC AAA CAC C | 54 | 175 |
|  | ND4_rev2* | GGG GAA GGG CTA TRT TTA |  |  |
| ND4 fragment 6 | ND4_for13 | GAA CGA ACC CAT AAC CGA A | 52 | 104 |
|  | ND4_rev13 | CAA TGA TTG TCA GTT CTC CTA T |  |  |
| ND4 fragment 7 | ND4_for3* | TAC AAA CCY TAY TAC CCC | 54 | 176 |
|  | ND4_rev3* | GTT CCY CCT CAY TGT GTA GA |  |  |
| ND4 fragment 8 | ND4_for8 | CCC TCT GAT GAT TAT TAG C | 56 | 95 |
|  | ND4_rev8 | TGC CTA GYC CRG TTA ATA |  |  |
| ND4 fragment 9 | ND4_for14 | TCA TTG CAT CCT TAT TCA ACT GAG | 52 | 110 |
|  | ND4_rev14 | GTG GGA GGG GGT GAT AGT |  |  |
| ND4 fragment 10 | ND4_for10 | TAA TCT CAG CCC TCT ACT | 58 | 83 |
|  | ND4_rev10 | TGG AGT GTT ATG ATT AGG TG |  |  |
| ND4 fragment 11 | ND4_for18 | CTA CAC AGT GAG GGG GAA C | 58 | 72 |
|  | ND4_rev18 | ACT AAC GAG ATG GTT ATA AGT TG |  |  |
| ND4 fragment 12 | ND4_for11* | CAC CTA ATC ATA ACA CTC CA | 58 | 62 |
|  | ND4_rev11* | GCT CAC ATT CTA ATG TTT GTT G |  |  |
| ND4 fragment 13 | ND4_for15 | TAC TAA TAG TAA AAC CCC AAC TTA | 56 | 106 |
|  | ND4_rev15 | GTG TAG TCT CAG GGT TAG |  |  |
| ND4 fragment 14 | ND4_for9* | ACA AAC ATT AGA ATG TGA GCC TA | 52 | 104 |
|  | ND4_revP5* | AAA GCC ACA GGA TTA CTT CT |  |  |
