# Supplementary material for: One Extinct Turtle Species Less: Pelusios seychellensis Is Not Extinct, It Never Existed
Source: PLoS One. 2013 Apr 3;8(4):e57116. doi: 10.1371/journal.pone.0057116 (PMC3616038; doi:10.1371/journal.pone.0057116)
Supplement: Table S2 — Partitioning scheme and models selected by the Bayesian Information Criterion in JMODELTEST 0.1.1 [59] . (DOC) [file pone.0057116.s002.doc]

**Table S2.** Partitioning scheme and models selected by the Bayesian Information Criterion in JMODELTEST 0.1.1 [59].

| **Partition** | **Position within alignment**  **(fragment length)** | **Model** | **Nst** | **TRatio** | **Rates** | **Shape** | **Pinvar** |
| --- | --- | --- | --- | --- | --- | --- | --- |
| 12S | 1–391 (391 bp) | TIM2+G | 6 | – | gamma | 0.2310 | 0 |
| cyt *b* | 392–1186 (795 bp) | HKY+I+G | 2 | 6.3031 | gamma | 1.3310 | 0.4720 |
| ND4 | 1187–1859 (673 bp) | TPM3uf+I+G | 6 | – | gamma | 1.1900 | 0.4540 |
| tRNA-His | 1860–1934 (75 bp) | TPM2uf+G | 6 | – | gamma | 0.4760 | 0 |
| Non-annotated | 1935–2013 (79 bp) | TPM2+G | 6 | – | gamma | 0.6240 | 0 |
| tRNA-Leu | 2014–2054 (41 bp) | TPM1 | 6 | – | equal | – | 0 |

For identity of the non-annotated DNA sequence between the DNA coding for tRNA-His and tRNA-Leu, see [26].
